# Potential Targets for Scalp Acupuncture and Brain Stimulation in Mental Disorders: Evidence From Large-Scale Meta-Analyses

**DOI:** 10.31083/AP45940

**Published:** 2026-06-28

**Authors:** Yuanyuan Li, Yuan Feng, Yuefeng Wu, Qiao Kong, Binlong Zhang, Yu Liu, Siyi Yu, Jiao Liu, Jin Cao, Pauline Jeong, Fangyuan Cui, Jian Kong

**Affiliations:** ^1^Department of Psychiatry, Massachusetts General Hospital, Harvard Medical School, Charlestown, MA 02129, USA; ^2^Department of Acupuncture, Guang’anmen Hospital, China Academy of Chinese Medical Sciences, 100053 Beijing, China; ^3^Acupuncture-Moxibustion and Tuina School, Chengdu University of Traditional Chinese Medicine, 611137 Chengdu, Sichuan, China; ^4^School of Traditional Chinese Medicine, Capital Medical University, 100069 Beijing, China; ^5^School of Life Sciences, Beijing University of Chinese Medicine, 100029 Beijing, China; ^6^Department of Neurology, Dongzhimen Hospital, Beijing University of Chinese Medicine, 100700 Beijing, China

**Keywords:** acupuncture therapy, neuroimaging, meta-analysis, mental disorders, transcranial direct current stimulation

## Abstract

**Background:**

Scalp acupuncture and brain stimulation techniques, such as transcranial electrical stimulation, have shown promise in the management of mental disorders. However, the identification of precise stimulation targets remains a critical bottleneck. Pinpointing brain regions associated with mental disorders is essential for optimizing the efficacy of these scalp-based therapies.

**Methods:**

Using Neurosynth Compose, we performed large-scale coordinate-based meta-analyses to identify potential targets for nine mental disorders: schizophrenia, bipolar disorder, major depressive disorder, anxiety disorders, obsessive-compulsive disorder, post-traumatic stress disorder, insomnia, autism spectrum disorder, and attention deficit hyperactivity disorder. The identified brain clusters were projected onto the scalp surface and spatially integrated with standard acupoints, and the electroencephalography (EEG) 10–20 system to define accessible intervention sites.

**Results:**

The analyses successfully delineated scalp targets for each of the nine investigated disorders. These findings largely corroborated existing clinical protocols while also suggesting novel regions as potential therapeutic targets. The results revealed both disorder-specific targets and cortical areas implicated across multiple psychiatric conditions. Notably, the dorsolateral and ventrolateral prefrontal cortices, middle temporal gyrus, and inferior parietal lobule emerged as critical overlapping targets, offering potential avenues for scalp acupuncture and brain stimulation in comorbid mental disorders.

**Conclusions:**

We propose refined, evidence-based protocols for scalp acupuncture and brain stimulation. These findings may advance precision-targeted neuromodulation strategies and support the development of novel therapeutic approaches in psychiatric care.

## Main Points

1. Large-scale meta-analyses identified evidence-based scalp targets for nine major mental disorders.

2. Disorder-specific cortical targets were mapped onto accessible scalp locations by integrating brain imaging findings with acupoints, and the electroencephalography (EEG) 10–20 system.

3. The dorsolateral prefrontal cortex, ventrolateral prefrontal cortex, middle temporal gyrus, and inferior parietal lobule serve as critical, overlapping neural targets across multiple mental disorders.

4. The identified targets largely support existing clinical scalp acupuncture and brain stimulation protocols while also suggesting novel intervention regions.

5. These findings provide a framework for more precise, evidence-based scalp acupuncture and neuromodulation strategies in psychiatric care.

## 1. Introduction

Transcranial stimulation techniques have gained substantial interest among researchers and clinicians in recent years. Within this domain, scalp acupuncture, particularly scalp electroacupuncture, has demonstrated its effectiveness in managing diverse mental disorders, such as autism, depression, and cognitive impairment [1, 2]. The selection of scalp acupuncture targets is thought to rely on the anatomical alignment between scalp topography and the underlying cerebral cortex, highlighting the necessity of pinpointing the precise neural substrates that govern therapeutic efficacy [3].

In long-standing clinical practice, scalp acupuncture protocols have been organized around the concept of “scalp lines”. While the World Health Organization (WHO) and China have standardized the nomenclature for these lines [4], their guidelines primarily define general localization and broad indications rather than precise and disorder-specific targets. Consequently, despite rapid progress in mapping the neural mechanisms of mental disorders, these neurobiological insights have not yet been systematically incorporated into standard scalp acupuncture protocols. Bridging this translational gap requires the precise identification of surface cortical regions implicated in the central pathophysiology of these disorders.

Recent advances in neuroimaging modalities, such as magnetic resonance imaging (MRI), electroencephalography (EEG), and magnetoencephalography (MEG), have vastly improved our understanding of the neuropathology of mental disorders. These advancements offer a unique opportunity to establish neuroscience-based targets, thereby refining the precision and clinical scope of scalp acupuncture.

The challenge of identifying optimal and evidence-based targets is not unique to scalp acupuncture. In parallel, other transcranial stimulation techniques, such as transcranial magnetic stimulation (TMS) and transcranial electrical stimulation (tES), have been gaining popularity in recent decades. While these modalities, including scalp acupuncture, possess distinct biophysical mechanisms of action, they all share a common therapeutic goal, that is, to modulate the activity of certain cortical regions associated with a specific disorder. To date, target selection for all these methods has often relied on traditional protocols or a limited number of studies. This highlights the need for a unified, large-scale, data-driven framework. Neuroimaging-informed and meta-analytic approaches have been increasingly used to provide this evidence base, offering a path to improve therapeutic outcomes [5, 6].

Neuroimaging meta-analyses provide a powerful approach for synthesizing evidence to inform target selection. However, due to the vast volume of existing literature, extracting and analyzing relevant data makes manual synthesis prohibitive. To address this challenge, we utilized Neurosynth Compose [7], a natural language processing–based platform that automates literature retrieval, text classification, and coordinate extraction, thereby enabling large-scale and coordinate-based meta-analyses by integrating data from multiple imaging modalities. While our earlier work [8, 9] clustered scalp stimulation targets, recent methodological advances, particularly in automated text mining and coordinate synthesis, allow for more comprehensive and reproducible analyses. These developments, along with the growing body of literature, necessitate re-clustering existing findings and identifying novel targets for intervention. For instance, our previous work [8, 9] included 77 publications on major depression, whereas the current study incorporates 835.

In this study, we focus on nine mental disorders: schizophrenia (SZ), bipolar disorder (BD), major depressive disorder (MDD), anxiety disorder (AD), obsessive-compulsive disorder (OCD), post-traumatic stress disorder (PTSD), insomnia, autism spectrum disorder (ASD), and attention deficit hyperactivity disorder (ADHD). Our primary objective is to bridge the gap between foundational neuroimaging findings and clinical practice for scalp-based therapies. To achieve this broad clinical goal, we employ a large-scale and data-driven computational approach. Specifically, we use machine learning, data mining, and coordinate-based meta-analysis to systematically synthesize the vast neuroimaging literature using Neurosynth Compose. This data-driven methodology allows us to move beyond single studies or traditional protocols to identify robust and evidence-based cortical targets. We then translate these neuroimaging-derived targets into practical protocols. We hope this work lays the foundation for new insights into therapeutic strategies and advances the field of scalp acupuncture and brain stimulation.

## 2. Materials and Methods

This study aims to identify precise cortical targets for scalp acupuncture and brain stimulation across various mental disorders. To achieve this, we employed Neurosynth Compose (https://compose.neurosynth.org), a next-generation platform for automated meta-synthesis, to systematically map brain surface targets from extensive literature [7, 10]. These neuroimaging findings were subsequently triangulated with the anatomical distribution of scalp acupoints to derive optimized and clinically applicable intervention strategies. Although this systematic review was not formally registered, it was conducted in accordance with the Preferred Reporting Items for Systematic Reviews and Meta-Analyses (PRISMA) 2020 guidelines [11]. The detailed study protocol, comprising the full search syntax, pre-defined inclusion/exclusion criteria, and analysis plan, and the PRISMA checklist are provided in the **Supplementary Materials**.

### 2.1 Literature Screening and Coordinate Information Organization

#### 2.1.1 Data Source and Search Strategy

Data acquisition was executed through Neurosynth Compose [7], a sophisticated environment designed for reproducible neuroimaging meta-synthesis. To guarantee both the breadth and accuracy of our dataset, we deployed a hybrid strategy that merged automated retrieval with manual curation. A systematic search was initialized within the platform to capture literature on target mental disorders published through November 12th, 2024. We accessed the underlying study data via the “Import via NeuroStore” utility. Functioning as a centralized open-science database for this field, NeuroStore contains approximately 40,000 studies. It aggregates content from multiple sources, including PubMed Central, core neuroimaging journals, NeuroQuery, and the original Neurosynth database [7].

The retrieved publications underwent independent eligibility screening by a panel of nine reviewers, strictly adhering to predefined inclusion and exclusion protocols. In instances of ambiguity, an additional independent reviewer adjudicated the decision to ensure consensus.

The targeted disorders included SZ, BD, MDD, AD, OCD, PTSD, insomnia, ASD, and ADHD. (1) “Schizophrenia”, “split personality disorder”, “paranoid schizophrenia” and “schizoaffective disorder” were used as search terms for SZ; (2) “Bipolar disorder”, “manic-depressive illness”, “manic depression” and “bipolar affective disorder” were used as search terms for BD; (3) “Major depression”, “clinical depression”, “major depressive disorder”, “unipolar depression” and “severe depression” were used as search terms for MDD; (4) “Anxiety disorder” was used as a search term for AD; (5) “Obsessive-compulsive disorder”, “OCD”, “compulsive behavior disorder” and “obsession disorder” were used as search terms for OCD; (6) “Post-traumatic stress disorder”, “PTSD”, “combat stress” and “post-trauma syndrome” were used as search terms for PTSD; (7) “Insomnia”, “sleeplessness”, “sleep deprivation”, “sleep disorder” and “difficulty sleeping” were used as search terms for insomnia; (8) “Autism spectrum disorder”, “autism”, “asperger syndrome”, “pervasive developmental disorder”, “childhood disintegrative disorder”, “high-functioning autism” and “low-functioning autism” were used as search terms for ASD; (9) “ADHD”, “attention deficit hyperactivity disorder”, “attention deficit disorder with hyperactivity”, “hyperkinesis” and “attention deficit and disruptive behavior disorders” were used as search terms for ADHD.

#### 2.1.2 Inclusion and Exclusion Criteria

Studies were deemed eligible for inclusion if they satisfied the following four criteria: (1) Recruitment of participant cohorts with a clinically confirmed diagnosis of the targeted mental disorders; (2) Implementation of experimental designs that involved direct contrasts between patient groups and healthy controls, or between intervention and control arms; (3) Employment of established neuroimaging modalities, including MRI, positron emission tomography, single-photon emission computed tomography, arterial spin labeling, EEG, or MEG; (4) Reporting of significant between-group effects using standard 3D coordinates in either Talairach or Montreal Neurological Institute (MNI) space.

Conversely, the following exclusion criteria were applied: (1) Investigations limited to healthy control groups or those relying solely on experimental models without clinical patients; (2) Research where the specific mental disorder was not the primary subject of inquiry, such as cases where symptoms were secondary to other pathologies or overshadowed by complex comorbidities; (3) Datasets failing to report coordinates in a standardized space; (4) Preclinical research utilizing animal models; (5) Computational or machine learning analyses focused on classification or treatment prediction rather than the localization of neural substrates; (6) Secondary literature, including prior meta-analyses, reviews, and single-subject case reports.

#### 2.1.3 Data Transparency

To promote open science and ensure the long-term utility of these findings, all datasets have been archived with stable and persistent digital identifiers. Because the fundamental objective of this research was to synthesize topological activation patterns for neuromodulation targeting, rather than to quantify the magnitude of clinical effects, a traditional risk of bias assessment was not conducted. However, rigorous quality control was maintained through our manual screening process, which excluded studies with non-standard coordinates, inappropriate designs, or insufficient reporting, ensuring that only high-quality, spatially precise data contributed to the analysis.

### 2.2 Automated Meta-Analysis Procedures

The coordinate-based meta-analysis was performed using the Multilevel Kernel Density Analysis with the Chi-Square (MKDAChi2) algorithm, a permutation-based and non-parametric procedure implemented in Neurosynth Compose supported by NiMARE. This method evaluates functional specificity using Study Contrast Maps as the unit of analysis, addressing the fixed-effects issue and enhancing generalizability [12]. This provides a rigorous test for the probability of activating a region, ensuring specific conclusions independent of overall activation frequency. The resulting uniformity test map for each disorder was corrected for multiple comparisons using the False Discovery Rate (FDR) at a *p* < 0.05.

### 2.3 Identifying Brain Targets From Neuroimaging Meta-Analyses

Scalp acupuncture and neuromodulation methods such as tES, primarily involve targeting the surface cerebral cortex. Thus, a standard cortical brain template (within 2.5 cm of the scalp) [13] was applied to the FDR corrected uniformity test map to identify cortically accessible brain areas. To identify a manageable number of primary targets for clinical translation, we employed a standardized thresholding procedure using DPABI version 8.1 (http://rfmri.org/dpabi). Starting with the FDR-corrected uniformity test map, we iteratively increased the t-value threshold in increments of 0.5. We continued this adjustment until 3 to 9 distinct clusters were identified, each with a voxel count between 30 and 900. This approach, consistent with our prior clinical-translational work [8, 9], allowed us to focus the protocol on the most statistically robust clusters (those with the highest peak t-values) for practical application. Crucially, this iterative process also functioned as a statistical segmentation strategy for clusters extending across multiple brain areas: by systematically raising the threshold, we isolated individual peak coordinates within large, confluent volumes, ensuring that clinical protocols target the distinct local maxima of activation. The full statistical maps, which contain many more clusters, are available for inspection in **Supplementary Tables 1–9**. The peak MNI coordinates of these clusters in AAL3 template [14] were reported using the xjView toolbox 10.0 (http://www.alivelearn.net/xjview/). The results were then mapped onto a standard brain using Surf Ice (https://www.nitrc.org/projects/surfice/) and a standard head using MRIcroGL (https://www.nitrc.org/projects/mricrogl/).

### 2.4 Identification of Targets and Needle Application Strategies

**Fig. 1.**
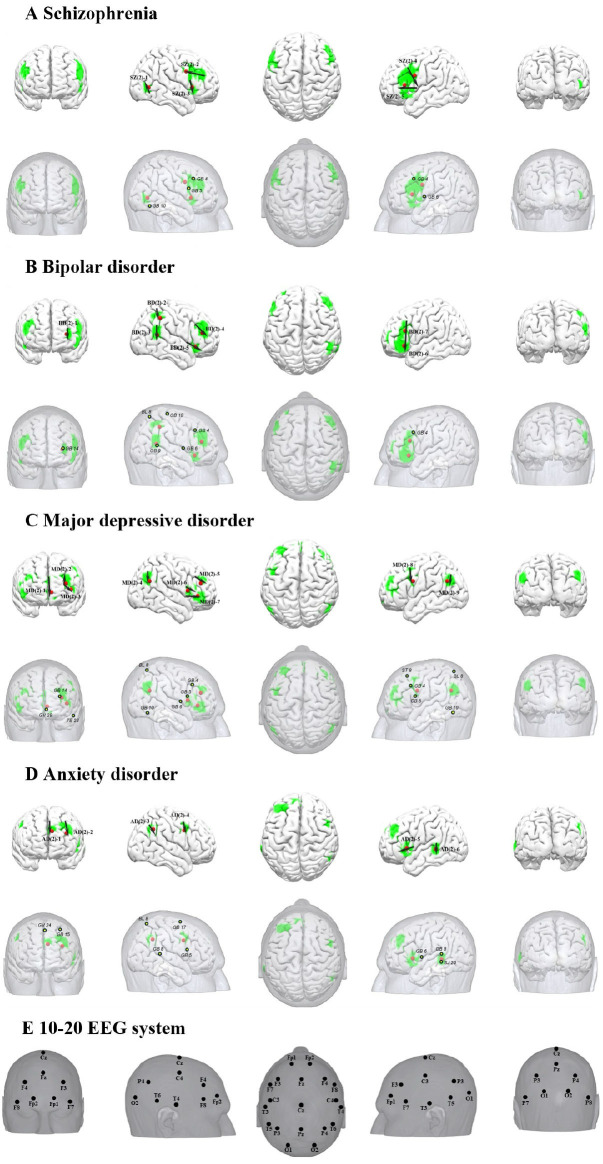
**Brain surface targets for scalp acupuncture for mental disorders 1–4**. (A) Schizophrenia related targets and suggested scalp acupuncture protocols. (B) Bipolar disorder related targets and suggested scalp acupuncture protocols. (C) Major depressive disorder related targets and suggested scalp acupuncture protocols. (D) Anxiety disorder related targets and suggested scalp acupuncture protocols. (E) Schematic diagram of the 10–20 EEG system. The brain and head models were rendered using Canvas (https://app.canvasenvision.com/), Surf Ice (https://www.nitrc.org/projects/surfice/) and MRIcroGL (https://www.nitrc.org/projects/mricrogl/) by the authors. EEG, electroencephalography.

**Fig. 2.**
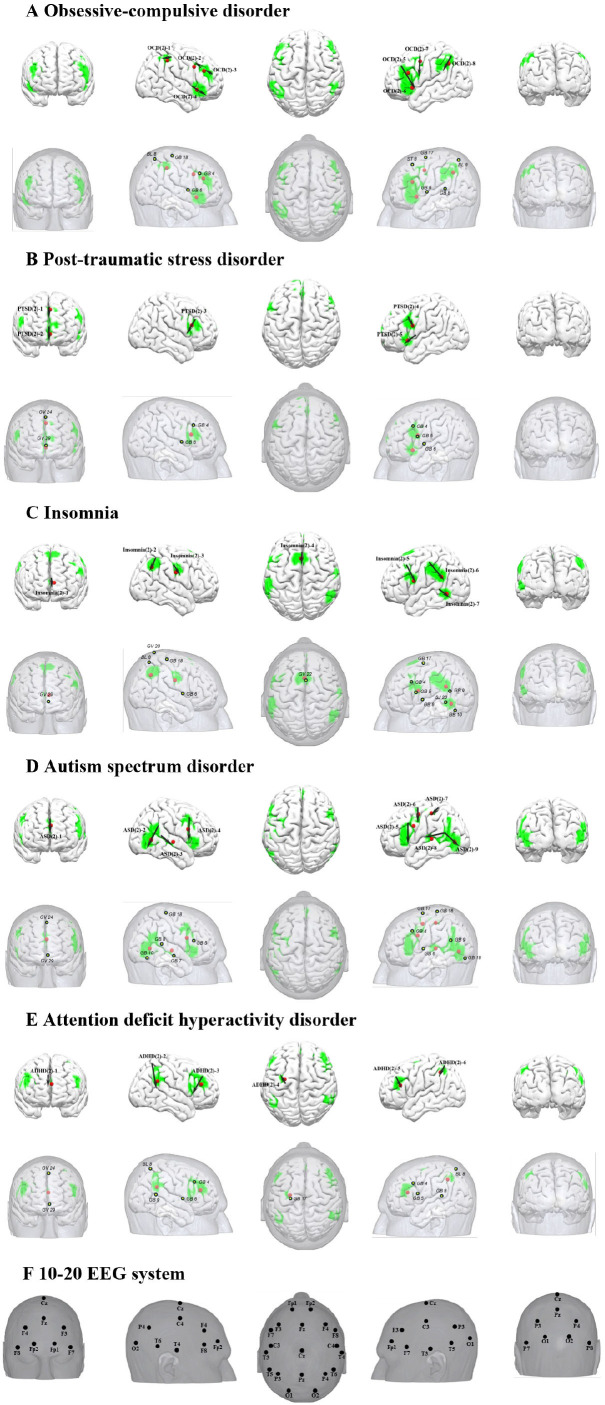
**Brain surface targets for scalp acupuncture for mental disorders 5–9**. (A) Obsessive-compulsive disorder related targets and suggested scalp acupuncture protocols. (B) Post-traumatic stress disorder related targets and suggested scalp acupuncture protocols. (C) Insomnia related targets and suggested scalp acupuncture protocols. (D) Autism spectrum disorder related targets and suggested scalp acupuncture protocols. (E) Attention deficit hyperactivity disorder related targets and suggested scalp acupuncture protocols. (F) Schematic diagram of the 10–20 EEG system. The brain and head models were rendered using Canvas (https://app.canvasenvision.com/), Surf Ice (https://www.nitrc.org/projects/surfice/) and MRIcroGL (https://www.nitrc.org/projects/mricrogl/) by the authors.

To precisely map the identified peak points onto the scalp surface, we employed the dual reference frameworks of the International 10–20 EEG system (Fig. 1E, Fig. 2F) and the WHO Proposed Standard International Acupuncture Nomenclature (1991). Going beyond simple surface localization, we analyzed the 3D volumetric geometry of each target brain cluster to engineer specific needle insertion vectors and manipulation strategies designed to maximize the engagement of the underlying neural tissue. A detailed schematic illustrating the relevant scalp acupoints and lines is available in **Supplementary Fig. 1**.

## 3. Results

The bibliometric workflow and the resultant meta-analytic statistics for each mental disorder are detailed in Table 1 and **Supplementary Fig. 2**. The corresponding scalp topography, including specific acupoints and lines, is visualized in **Supplementary Fig. 1**, and the complete whole-brain coordinate data derived from the meta-analyses are cataloged in **Supplementary Tables 1–9**. To ensure the protocols are accessible across diverse linguistic and clinical contexts, **Supplementary Table 10** provides a comparative nomenclature index (International, Chinese, Japanese, and Korean) for all relevant acupoints. Finally, to clearly distinguish novel data from our prior investigations [8, 9], targets associated with disorders previously analyzed are explicitly tagged with the suffix “(2)”.

**Table 1.** **Details of the inclusion process and final image results**.

Disease	Number of retrieved publications	Number of included publications	Number of analyses	Number of coordinates	Data identifier and view
SZ	1598	946	1576	23,764	https://identifiers.org/neurovault.collection:18473
BD	486	325	495	6233	https://identifiers.org/neurovault.collection:18474
MDD	1643	835	1209	13,781	https://identifiers.org/neurovault.collection:18452
AD	705	223	354	4435	https://identifiers.org/neurovault.collection:18480
OCD	293	231	364	4858	https://identifiers.org/neurovault.collection:18436
PTSD	317	258	510	6017	https://identifiers.org/neurovault.collection:18435
Insomnia	295	71	98	759	https://identifiers.org/neurovault.collection:18447
ASD	614	475	789	12,705	https://identifiers.org/neurovault.collection:18481
ADHD	375	237	400	5349	https://identifiers.org/neurovault.collection:18681

Abbreviations: SZ, schizophrenia; BD, bipolar disorder; MDD, major depressive disorder; AD, anxiety disorder; OCD, obsessive-compulsive disorder; PTSD, post-traumatic stress disorder; ASD, autism spectrum disorder; ADHD, attention deficit hyperactivity disorder.

### 3.1 Schizophrenia (SZ)

The meta-analysis identified five potential scalp acupuncture targets for SZ. The locations, recommended interventions, and corresponding brain regions for these targets are depicted in Fig. 1A and Table 2. Targets 2 and 3 were derived from the spatial segmentation of a single extensive cluster. A similar segmentation process was applied to separate Targets 4 and 5.

**Table 2.** **Targets in surface regions identified from the meta-analysis and scalp acupuncture strategies for mental disorders 1–4**.

Targets		Number of voxels	T value	Peak MNI			Position and operation suggestions	Corresponding brain area
				X	Y	Z		
SZ (2)								
	1	33	7.77	48	–66	2	GB10, posterior-superior transverse insertion (R)	Middle temporal gyrus (R)
	2	752	13.30	46	8	32	Midpoint of the line from GB4 to GB5, posterior transverse insertion (R)	Precentral gyrus (R)
								Inferior/middle frontal gyrus (R)
	3			50	20	2	GB5, anterior-inferior transverse insertion (R)	[LPFC/DLPFC (R)]
	4	856	16.58	–44	10	30	GB4 to GB6, posterior-inferior transverse insertion (L)	Precentral gyrus (L)
								Inferior/middle frontal gyrus (L)
								Superior temporal gyrus (L)
								Rolandic operculum (L)
	5			–50	18	–4	GB6, anterior transverse insertion (L)	[LPFC/DLPFC (L)]
BD (2)								
	1	46	4.19	–28	52	10	GB14, inferior transverse insertion (L)	Middle/superior frontal gyrus (L)
								[LPFC/DLPFC (L)]
	2	128	5.17	48	–44	46	Midpoint of the line from BL8 to GB18, inferior transverse insertion (R)	Inferior parietal gyrus(R)
								Supramarginal gyrus (R)
								Angular gyrus (R)
	3	144	5.50	58	–50	14	∼2 cm superior to GB9, inferior transverse insertion (R)	Middle/superior temporal gyrus (R)
								Supramarginal gyrus (R)
								Angular gyrus (R)
	4	390	5.50	46	40	18	GB4, anterior-inferior transverse insertion (R)	Middle/inferior frontal gyrus (R)
								[LPFC/DLPFC (R)]
	5	50	5.82	48	26	–8	GB6, anterior-inferior transverse insertion (R)	Inferior frontal gyrus (R)
								Posterior orbital gyrus (R)
								[LPFC (R)]
	6	350	7.10	–46	28	–6	GB4, inferior transverse insertion (L)	Inferior/middle frontal gyrus (L)
								Lateral orbital gyrus (L)
	7			–50	26	22		[LPFC/DLPFC (L)]
MDD (2)								
	1	358	9.09	–4	58	0	GV29, inferior transverse insertion	Medial prefrontal gyrus (Bil)
	2	149	5.24	–32	52	18	GB14, superior transverse insertion (L)	Middle/superior/inferior frontal gyrus (L)
								[LPFC/DLPFC (L)]
	3	31	5.02	–42	38	6	Upper 1/2 of the line from GB14 to TE23, posterior-inferior transverse insertion (L)	Inferior frontal gyrus (L)
								[LPFC (L)]
	4	238	6.73	50	–60	28	Middle 1/3 of the line from BL8 to GB10, inferior transverse insertion (R)	Angular gyrus (R)
								Middle occipital gyrus (R)
								Inferior parietal gyrus(R)
	5	140	5.66	38	42	24	GB4, anterior-inferior transverse insertion (R)	Middle/superior/inferior frontal gyrus (R)
								[LPFC/DLPFC (R)]
	6	35	4.81	56	16	10	GB5, anterior-inferior transverse insertion (R)	Inferior frontal gyrus (R)
								[LPFC (R)]
	7	80	5.02	48	36	0	GB6, anterior transverse insertion (R)	Inferior/middle frontal gyrus (R)
								[LPFC (R)]
	8	144	5.45	–48	6	28	∼1 cm posterior to ST8, transverse insertion toward GB5 (L)	Inferior / middle frontal gyrus (L)
								Precentral gyrus (L)
								[LPFC/DLPFC (L)]
	9	117	5.87	–50	–62	28	Middle 1/3 of the line from BL8 to GB10, inferior transverse insertion (L)	Angular gyrus (L)
AD (2)								
	1	62	3.11	–6	54	30	GV24, inferior transverse insertion	Superior frontal gyrus (L)
								[LPFC/DLPFC (L)]
	2	279	4.72	–34	38	26	GB15, anterior-inferior transverse insertion (L)	Middle/superior/inferior frontal gyrus (L)
								[LPFC/DLPFC (L)]
	3	62	4.32	48	–52	38	Middle 1/3 of the line from BL8 to GB8, anterior-inferior transverse insertion (R)	Inferior parietal gyrus (R)
	4	87	3.92	48	10	36	Middle 1/3 of the line from GB17 to GB5, anterior-inferior transverse insertion (R)	Precentral gyrus (R)
								Inferior frontal gyrus (R)
								[LPFC (R)]
	5	72	5.11	–50	18	2	GB6, anterior-inferior transverse insertion (L)	Inferior frontal gyrus(L)
								Superior temporal gyrus (L)
								[LPFC (L)]
	6	31	3.52	–62	–40	1	GB8 to SJ20, inferior transverse insertion (L)	Middle temporal gyrus (L)

Abbreviations: SZ, schizophrenia; BD, bipolar disorder; MDD, major depressive disorder; AD, anxiety disorder; MNI, Montreal Neurological Institute; L, left; R, right; Bil, bilateral; LPFC, lateral prefrontal cortex; DLPFC, dorsolateral prefrontal cortex.

### 3.2 Bipolar Disorder (BD)

The meta-analysis identified seven potential scalp acupuncture targets for BD. The locations, recommended interventions, and corresponding brain regions for these targets are depicted in Fig. 1B and Table 2. Targets 6 and 7 were derived from the spatial segmentation of a single extensive cluster.

### 3.3 Major Depressive Disorder (MDD)

The meta-analysis identified nine potential scalp acupuncture targets for MDD. The locations, recommended interventions, and corresponding brain regions for these targets are depicted in Fig. 1C and Table 2.

### 3.4 Anxiety Disorder (AD)

The meta-analysis identified six potential scalp acupuncture targets for AD. The locations, recommended interventions, and corresponding brain regions for these targets are depicted in Fig. 1D and Table 2.

### 3.5 Obsessive-Compulsive Disorder (OCD)

The meta-analysis identified eight potential scalp acupuncture targets for OCD. The locations, recommended interventions, and corresponding brain regions for these targets are depicted in Fig. 2A and Table 3. Targets 5 and 6 were derived from the spatial segmentation of a single extensive cluster.

**Table 3.** **Targets in surface regions identified from the meta-analysis and scalp acupuncture strategies for mental disorders 5–9**.

Targets		Number of voxels	T value	Peak MNI			Position and operation suggestions	Corresponding brain area
				X	Y	Z		
OCD (2)								
	1	101	5.06	46	–40	48	∼1 cm inferior to the line from GB18 to BL8, posterior-inferior transverse insertion (R)	Inferior parietal gyrus(R)
								Supramarginal gyrus (R)
								Postcentral gyrus (R)
	2	39	3.91	42	14	36	Lower 1/3 of the line from GB4 to GB18, posterior-superior transverse insertion (R)	Inferior/middle frontal gyrus (R)
								Precentral gyrus (R)
								[LPFC (R)]
	3	339	4.68	40	34	28	On the extension line between GB18 and GB4, ∼2 cm inferior to GB4, transverse insertion toward GB4 (R)	Middle/inferior frontal gyrus (R)
								[LPFC/DLPFC (R)]
	4	199	5.82	52	20	–8	GB6, anterior-inferior transverse insertion (R)	Inferior frontal gyrus (R)
								Superior temporal gyrus (R)
								Lateral/posterior orbital gyrus (R)
								[LPFC (R)]
	5	709	6.59	–42	26	24	∼1 cm anterior-inferior to the line from ST8 to GB6, posterior-inferior transverse insertion (L)	Inferior frontal gyrus (L)
								Precentral gyrus (L)
	6			–50	18	–4		[LPFC/DLPFC (L)]
	7	142	4.30	–38	2	46	GB17 to GB6, inferior transverse insertion (L)	Precentral gyrus (L)
								Middle/inferior frontal gyrus (L)
								[LPFC (L)]
	8	251	4.68	–42	–56	42	BL8 to GB8, anterior-inferior transverse insertion (L)	Angular gyrus (L)
								Inferior parietal gyrus(L)
								Supramarginal gyrus (L)
PTSD (2)								
	1	58	3.99	0	36	46	GV24, inferior transverse insertion	Medial prefrontal gyrus (Bil)
	2	394	6.36	0	58	–4	GV29, inferior transverse insertion	Inferior/superior frontal gyrus (L)
								Medial prefrontal gyrus (Bil)
								[LPFC/DLPFC (L)]
	3	337	6.02	48	22	20	∼0.5 cm anterior to the line from GB4 to GB6, posterior-inferior transverse insertion (R)	Inferior/middle frontal gyrus (R)
								Precentral gyrus (R)
								[LPFC/DLPFC (R)]
	4	188	4.33	–52	8	22	GB4 to GB5, posterior-inferior transverse insertion (L)	Inferior/middle frontal gyrus (L)
								Precentral gyrus (L)
								[LPFC/DLPFC (L)]
	5	43	6.02	–50	18	–6	GB6, anterior-inferior transverse insertion (L)	Inferior frontal gyrus (L)
								Superior temporal gyrus (L)
								[LPFC (L)]
Insomnia								
	1	35	2.83	–6	56	14	GV29, inferior transverse insertion	Medial prefrontal gyrus (Bil)
	2	164	3.74	52	–60	40	∼2 cm lateral-inferior transverse line from GV20 to BL8, posterior-inferior transverse insertion (R)	Inferior parietal gyrus (R)
								Angular gyrus (R)
	3	106	2.83	56	–12	30	Middle 1/3 of the line from GB18 to GB6, anterior-inferior transverse insertion (R)	Postcentral gyrus (R)
								Precentral gyrus (R)
								Supramarginal gyrus (R)
	4	332	6.51	0	20	58	GV22, posterior transverse insertion (L)	Supplementary motor area (Bil)
								Medial prefrontal gyrus (Bil)
	5	171	2.83	–52	6	22	GB4 to GB5, posterior-inferior transverse insertion (L)	Precentral gyrus (L)
								Inferior/middle frontal gyrus (L)
								[LPFC/DLPFC (L)]
	6	280	3.74	–62	–50	32	Lower 1/2 of the line from GB17 to GB9, posterior-inferior transverse insertion (L)	Supramarginal gyrus (L)
								Inferior parietal gyrus (L)
								Postcentral gyrus (L)
								Angular gyrus (L)
	7	86	2.83	–54	–60	–2	SJ20 to GB10, posterior-inferior transverse insertion (L)	Middle/inferior temporal gyrus (L)
ASD (2)								
	1	146	5.34	0	52	26	Lower 1/2 of the line from GV24 to GV29, inferior transverse insertion	Medial prefrontal gyrus (Bil)
	2	691	8.49	54	–60	4	Lower 1/2 of the line from GB18 to GB10, posterior-inferior transverse insertion (R)	Middle/superior/inferior temporal gyrus (R)
								Angular gyrus (R)
								Supramarginal gyrus (R)
								Middle/inferior occipital gyrus (R)
	3	51	6.24	58	–16	0	GB8 to GB7, anterior-inferior transverse insertion (R)	Superior temporal gyrus (R)
	4	463	7.82	48	14	24	GB5, inferior transverse insertion (R)	Inferior / middle frontal gyrus (R)
								Precentral gyrus (R)
								[LPFC/DLPFC (R)]
	5	497	7.60	–46	8	30	GB4, inferior transverse insertion (L)	Precentral gyrus (L)
								Inferior / middle frontal gyrus (L)
								[LPFC/DLPFC (L)]
	6	42	5.12	–32	–2	52	GB17, inferior transverse insertion (L)	Middle/superior frontal gyrus (L)
								Precentral gyrus (L)
	7	32	5.57	–42	–28	54	∼1 cm posterior to GB18, anterior-inferior transverse insertion (L)	Postcentral gyrus (L)
								Inferior parietal gyrus (L)
	8	34	5.34	–60	–28	6	GB9 to GB6, anterior-inferior transverse insertion (L)	Superior/middle temporal gyrus (L)
	9	519	6.92	–42	–74	0	GB9 to GB19, posterior-inferior transverse insertion (L)	Middle/inferior occipital gyrus (L)
								Middle/inferior temporal gyrus (L)
								Angular gyrus (L)
ADHD (2)								
	1	39	4.23	0	54	20	Lower 1/2 of the line from GV24 to GV29, inferior transverse insertion	Medial prefrontal gyrus (Bil)
	2	258	4.58	56	–48	28	Lower 2/3 of the line from BL8 to GB9, anterior-inferior transverse insertion (R)	Supramarginal gyrus (R)
								Inferior parietal gyrus(R)
								Angular gyrus (R)
								Superior temporal gyrus (R)
	3	738	5.63	38	38	22	GB4 to GB6, posterior-inferior transverse insertion (R)	Middle/inferior frontal gyrus (R)
								Precentral gyrus (R)
								[LPFC/DLPFC (R)]
	4	30	4.23	–26	–6	60	GB17, anterior-inferior transverse insertion (L)	Superior/middle frontal gyrus (L)
								Precentral gyrus (L)
	5	170	4.23	–44	30	20	∼0.5 cm anterior to the line from GB4 to GB5, posterior-inferior transverse insertion (L)	Inferior/middle frontal gyrus (L)
								[LPFC/DLPFC (L)]
	6	95	5.28	–42	–52	46	Middle 1/3 of the line from BL8 to GB8, anterior-inferior transverse insertion (L)	Inferior parietal gyrus (L)
								Angular gyrus (L)

Abbreviations: OCD, obsessive-compulsive disorder; PTSD, post-traumatic stress disorder; ASD, autism spectrum disorder; ADHD, attention deficit hyperactivity disorder; MNI, Montreal Neurological Institute; L, left; R, right; Bil, bilateral; LPFC, lateral prefrontal cortex; DLPFC, dorsolateral prefrontal cortex.

### 3.6 Post-Traumatic Stress Disorder (PTSD)

The meta-analysis identified five potential scalp acupuncture targets for PTSD. The locations, recommended interventions, and corresponding brain regions for these targets are depicted in Fig. 2B and Table 3.

### 3.7 Insomnia

The meta-analysis identified seven potential scalp acupuncture targets for insomnia. The locations, recommended interventions, and corresponding brain regions for these targets are depicted in Fig. 2C and Table 3.

### 3.8 Autism Spectrum Disorder (ASD)

The meta-analysis identified nine potential scalp acupuncture targets for ASD. The locations, recommended interventions, and corresponding brain regions for these targets are depicted in Fig. 2D and Table 3.

### 3.9 Attention Deficit Hyperactivity Disorder (ADHD)

The meta-analysis identified six potential scalp acupuncture targets for ADHD. The locations, recommended interventions, and corresponding brain regions for these targets are depicted in Fig. 2E and Table 3.

## 4. Discussion

By synthesizing data through large-scale neuroimaging meta-analyses, we have identified precise and evidence-based cortical targets for scalp acupuncture and brain stimulation across nine distinct mental disorders. These neuroanatomically grounded protocols refine existing therapeutic frameworks, offering a robust pathway to enhance precision and efficacy of neuromodulation therapies.

### 4.1 Core Brain Targets Across Mental Disorders

Our large-scale neuroimaging meta-analyses delineated key cortical targets associated with 9 mental disorders, with distinct neuroanatomical patterns for each condition.

In SZ, the inferior frontal gyrus, precentral gyrus, and middle temporal gyrus emerge as principal loci of abnormality. These areas are implicated in the neuropathology underlying cognitive deficits, auditory hallucinations, and other perceptual disturbances [15]. For mood disorders, including BD and MDD, the inferior frontal gyrus and middle frontal gyrus (dorsolateral prefrontal cortex, DLPFC) are prominently involved, accompanied by alterations in the middle temporal gyrus, inferior parietal lobule, and angular gyrus. This network is integral to emotion regulation, cognitive processing, and perception [16].

AD and OCD share key targets, such as the inferior frontal gyrus, inferior parietal lobule and precentral gyrus, which contribute to stress response and emotion regulation [17]. Nevertheless, each disorder also exhibits unique involvements. OCD is specifically linked to the angular gyrus, while AD shows distinct associations with the middle temporal gyrus, potentially reflecting their divergent clinical presentations.

PTSD is characterized by hyperactivation in the superior and inferior frontal gyri, patterns that correlate with heightened emotional reactivity [18] and the intrusive recall of traumatic memories [19]. Insomnia-related targets include the middle temporal gyrus, precentral gyrus, supplementary motor area, and inferior parietal gyrus, indicating contributions of motor regulation and sensory-emotional processing [20] in sleep pathology.

Among neurodevelopmental disorders, ADHD primarily involves the dorsolateral prefrontal cortex and inferior parietal lobule, regions critical for executive control and attention regulation [21]. In contrast, ASD is linked to the middle and superior temporal gyri, areas associated with language and social cognition, alongside frontal regions subserving higher-order cognitive functions [22].

In summary, this study provides a neuroimaging-based foundation for refining scalp acupuncture and neuromodulation targets by establishing a robust framework for understanding the distinct pathological mechanisms across various mental disorders.

### 4.2 Frequently Targeted Brain Regions and Their Functional Roles

Our analyses identified several cortical targets that show overlapping involvement across multiple mental disorders, suggesting a potential contribution to cognitive and affective processes that are commonly disrupted in these conditions.

The prefrontal cortex serves as a central hub for regulating cognitive and emotional processes and supporting adaptive behavior in complex environments [23]. The DLPFC, which includes regions of the superior and the middle frontal gyri (Brodmann areas 9 and 46), is primarily engaged in higher-order cognitive functions, including executive control, working memory, decision-making, and attention regulation [24]. These processes are critical for adaptive behavior, especially in complex or novel situations [25]. DLPFC dysfunction is a common feature across several mental disorders, including MDD [26], ADHD [27], and ASD [28].

The ventrolateral prefrontal cortex (VLPFC), encompassing the inferior frontal gyrus (Brodmann areas 44, 45, and 47), is another critical node for emotion and behavioral regulation, as well as language processing [29]. As a key component of the VLPFC, the inferior frontal gyrus collaborates closely with the DLPFC and ventromedial prefrontal cortex (VMPFC) to modulate emotional responses and inhibit impulsive actions [30]. Impairments within the inferior frontal gyrus have been widely observed in MDD [31], BD [32], and AD [33], which lead to challenges in cognitive flexibility [34], a diminished capacity to suppress inappropriate behaviors [35], and heightened emotional reactivity [36].

The precentral gyrus (Brodmann area 4), while traditionally linked to motor control, also contributes to sensorimotor integration and goal-directed behaviors [37]. Dysfunctions in this region are noted in ADHD [38], ASD [39], insomnia, and SZ [40], and may underlie the deficits in motor planning, behavioral regulation, and sensorimotor processing that characterize these disorders [41].

The temporal lobe, essential for social cognition, memory, and language, is frequently implicated in various mental disorders [42]. Specifically, the middle temporal gyrus (Brodmann areas 21 and 37) is altered in conditions such as AD [43], BD [44], and ASD [45]. This region supports social cognition, semantic processing, and language comprehension [46]. Its dysfunctions can manifest as social withdrawal, communication difficulties, and impaired interpretation of social cues, highlighting its importance in the overlapping neurobiology of these conditions [47].

The parietal lobe, essential for integrating sensory information and supporting higher-order cognitive functions, has been frequently implicated in mental disorders [48]. Particularly, the inferior parietal lobule (Brodmann areas 39 and 40) appears as a common locus in disorders like ADHD [49], OCD [50], and insomnia [51], where it contributes to attention, sensory integration, and self-referential processing [52]. Pathology changes in this area are linked to attentional control deficits, disrupted sensory processing, and impaired self-awareness, which may foster the cognitive disorganization and ruminative tendencies seen in these conditions [53].

The convergent involvement of these regions across diagnostic boundaries underscores their potential as neuromodulation targets for treating multiple disorders that may exhibit common pathophysiological mechanisms.

### 4.3 Comparative Analysis of Clinically Related Disorders

Several mental disorders present diagnostic challenges due to overlapping symptomatology and partially shared neural mechanisms. However, a closer examination of their neural correlates reveals distinct pathophysiological underpinnings.

Mood disorders BD and MDD serve as a prime example. Both disorders implicate the inferior frontal gyrus, a region pivotal for emotion regulation and impulse control [54]. In BD, aberrant activity in this area correlates with pronounced mood fluctuations, whereas in MDD, it relates more closely to persistent low mood and negative self-evaluation. Notably, BD involves a more extensive network including the middle frontal gyrus, middle temporal gyrus, and inferior parietal lobe, potentially reflecting the disorder’s complex emotional and cognitive disturbances, particularly during manic episodes. Conversely, MDD primarily engages the DLPFC and angular gyrus, regions more specifically tied to executive function and negative cognitive biases. These neuroanatomical distinctions align with their divergent clinical presentations.

Neurodevelopmental disorders ADHD and ASD also demonstrate both overlap and divergence. While both conditions involve attention deficits and social challenges [55], and share abnormalities in regions supporting cognitive processing and attention, such as the inferior frontal gyrus and inferior parietal lobule [56, 57], their primary neural signatures differ. ADHD is characterized by dysfunction in cognitive control networks, particularly the middle frontal gyrus and inferior frontal triangular part, suggesting disturbances in cognitive control, attention, and impulsivity [58]. In contrast, ASD shows stronger associations with regions governing social cognition and sensory integration, including the middle and superior temporal gyri, precentral gyrus, and postcentral gyrus. This pattern highlights ASD’s fundamental challenges in social communication and sensory processing. Thus, while ADHD’s neural correlates emphasize attentional and inhibitory control circuits [59], ASD’s profile centers on social-informative sensory integration networks.

Another diagnostically relevant pairing is AD and OCD. These conditions have common targets, including the inferior frontal gyrus, middle frontal gyrus, and inferior parietal lobule, regions subserving emotion regulation, cognitive control, and sensory processing. Both also involve the precentral gyrus (motor control) and superior medial frontal gyrus (emotional regulation). These commonalities may underlie shared features like anxiety, compulsive behaviors, and cognitive control difficulties. Nevertheless, subtle differences in the functional dynamics within these shared networks likely contribute to distinct clinical phenotypes.

By systematically comparing the neural architecture of clinically related disorders, we gain a more nuanced understanding of their overlapping and divergent pathophysiologies. These insights not only inform differential diagnosis but also pave the way for developing more precisely targeted neuromodulation protocols that address the unique neurobiological features of each condition.

### 4.4 Consistencies and Differences Between the Suggested Targets and Current Literature-Documented Treatment Targets

The cortical targets identified in our analysis demonstrate both meaningful convergence with established neuromodulation sites and distinctive features that may offer novel therapeutic insights.

For instance, TMS targeting the DLPFC is a well-established intervention for MDD [60], with this region regarded as central to the neurobiological mechanisms supporting clinical improvement [61]. Our findings corroborate this, showing extensive DLPFC engagement. Functional and structural alterations in the DLPFC are thought to reflect disrupted emotional processing, including biased evaluation of positive and negative stimuli [26]. Neuromodulation of this region may therefore help rebalance affective circuits and alleviate depressive symptoms.

In the domain of acupuncture, a prior meta-analysis [62] identified scalp acupoints along the midline, such as GV20, GV29, and GV24, as commonly employed for depression. Our proposed targets similarly emphasize GV29 and adjacent midline structures, which are traditionally associated with tranquilizing effects. Our meta-analytic targets also show strong convergence with other scalp acupuncture studies. For instance, our analysis identified the prefrontal and middle temporal cortices as key targets for AD. This aligns with protocols described by a case report [63], which targeted scalp zones corresponding to these underlying regions for anxiety. Furthermore, our identification of prefrontal and parietal targets for MDD and AD is consistent with the scalp targets used by others for post-stroke depression and anxiety [64]. This convergence suggests our data-driven approach can effectively confirm and refine targets used in clinical practice.

Notably, our protocol also extends beyond conventionally targeted areas. For instance, we identified symmetrical targets over the bilateral angular gyrus, anatomically proximate to acupoints BL8 and GB10. Traditionally, practitioners believe that stimulation in this vicinity enhances mental clarity and produces a refreshing effect. The angular gyrus is critically involved in episodic memory and semantic cognition [65], processes frequently compromised in depression. Modulating activity in these regions might facilitate improvements in memory storage and retrieval, potentially supporting more adaptive behavioral responses.

As anticipated, the targets identified here only partially overlap with those from our earlier investigations [8, 9]. These differences highlight the specific novelties of the current study. First, the significantly more advanced Neurosynth Compose platform allowed for more rigorous and reproducible study selection and analysis than the previous version. This enhanced framework permitted stricter inclusion and exclusion criteria and more selective extraction of relevant coordinate data to avoid the indiscriminate incorporation of all available findings. Second, the vastly larger and more current dataset provided a more robust statistical power, and the substantially larger number of publications analyzed in this study facilitated a more comprehensive and reliable synthesis. For instance, while our earlier work successfully identified core prefrontal targets, the current study uncovered additional posterior targets. The third distinction lies in the expanded diagnostic scope. The current study included comprehensive analyses for insomnia, which was not previously analyzed, filling a critical gap in the literature. Collectively, these methodological advancements contributed to the enhanced accuracy and robustness of the current results.

### 4.5 Comparison With Other Methods to Identify Targets for Brain Stimulation

Our results significantly converge with the findings of causal network-based studies. For instance, altered brain function and causal connectivity in the DLPFC circuit following repetitive TMS for MDD have been reported [66], which strongly corroborates our identification of the DLPFC as a primary target for MDD. Our identification of the medial prefrontal gyrus is also strongly supported by a causal network analysis of the rostral anterior cingulate network in depression [67].

Similarly, our findings of prefrontal and parietal involvement in SZ and OCD are consistent with other recent causal connectivity analyses [68, 69]. A causal network analysis study utilizing intracranial Local Field Potential signals demonstrated that patients hospitalized for invasive epilepsy monitoring exhibited stronger causal influence in effective networks, reflected by a significantly higher outdegree in the DLPFC during a cognitive control task [70]. This finding supports the DLPFC as a potential stimulation site for detecting and rectifying cognitive control deficits, with the aim of treating mental disorders. This convergence of evidence from different methodologies increases our confidence in the identified regions as robust targets for neuromodulation.

Beyond causal network analysis, other methods such as lesion-network mapping and stimulation-based approaches have also been applied to identify brain stimulation targets. These methods provide a valuable framework for pinpointing causal neuromodulation sites across mental disorders [71, 72]. By integrating data from lesions, TMS, and deep brain stimulation (DBS), this method can map symptom-specific networks that potentially cut across traditional diagnostic categories.

Applying these approaches to disorders such as SZ, MDD, PTSD, OCD, and AD has revealed several target regions that overlap with our meta-analytic results (**Supplementary Tables 11,12**). In MDD, overlapping areas include the DLPFC, VMPFC, and inferior frontal gyrus. For SZ, consistency is seen in the retrosplenial cortex and superior temporal gyrus. The medial prefrontal cortex has been commonly implicated in PTSD, while OCD shows overlap in the frontal pole and lateral/orbital prefrontal regions. Anxiety-related studies point to convergence in the right lateral parietal lobe. These overlaps highlight potential transdiagnostic neuromodulation targets and underscore the complementary nature of causal circuit mapping and meta-analytic methods [73, 74].

Nevertheless, lesion- and stimulation-derived circuit mapping may carry certain potential methodological limitations. First, these approaches typically rely on retrospectively collected data from case reports or clinical trials, which may vary in neuroimaging resolution, inclusion criteria, and symptom evaluation tools [74]. Second, they generally use normative connectome datasets derived from healthy individuals to infer functional circuits, which may not fully capture disease-specific or patient-specific connectivity patterns [73]. Third, heterogeneity in lesion etiology and variability in stimulation modalities complicate the interpretation of network findings across disorders [75]. Fourth, the use of diverse clinical scales and diagnostic systems may capture overlapping but non-equivalent constructs, posing challenges for cross-study integration [73].

In contrast, our coordinate-based meta-analysis synthesizes neuroimaging data collected directly from patient cohorts under pathological conditions. By aggregating reported activation coordinates and applying a uniform statistical framework, this method minimizes variability due to different analytic pipelines and identifies brain regions consistently associated with clinical symptoms. Rather than relying on normative connectivity inferences, Neurosynth Compose is grounded in the empirical distribution of patient-level neuroimaging abnormalities, providing a solid evidence base to contextualize and extend circuit models derived from lesion- or stimulation-based studies.

### 4.6 Deep Brain Structures

Our proposed protocols emphasize cortical targets due to the anatomical limitations of scalp acupuncture and most non-invasive brain stimulation techniques, which cannot directly access subcortical areas. Nevertheless, we acknowledge the established importance of deep brain structures in mental disorders. To ensure a comprehensive account and to support research involving methods capable of modulating these regions, we provide whole-brain meta-analytic results for each disorder in **Supplementary Tables 1–9**.

It is worth mentioning that some of the identified cortical areas can maintain strong functional and anatomical connections with deep brain regions. A key example is the amygdala, a structure strongly implicated in disorders such as PTSD and a potential target for achieving sustained therapeutic improvements through deep brain stimulation [76]. While the amygdala itself is beyond the direct reach of superficial techniques, the medial prefrontal cortex, a region corresponding to GV29, is well-documented to connect with the amygdala [77]. Stimulating this cortical site offers a viable and non-invasive strategy for modulating the amygdala-related circuits that underpin pathological fear and emotional responses.

### 4.7 Clinical Applicability and Feasibility

A critical component of this study is the reliable translation of complex volumetric neuroimaging data into usable scalp locations, ensuring clinical applicability. The challenge is converting statistically identified MNI coordinates into targets that clinicians can reliably locate without access to personalized fMRI or specialized neuronavigation.

The foundation of scalp acupuncture protocols rests on the principle of selecting acupoints that directly correspond to underlying brain regions [3]. To adhere to this principle and ensure high reproducibility in routine practice, we employ a dual-reference system: mapping the MNI coordinates onto the widely accepted WHO International Standard Scalp Acupuncture Nomenclature and the 10–20 EEG system. This dual-reference strategy ensures the protocols are highly feasible for integration into various real-world clinical settings, including community health centers, by providing anatomical landmarks locatable using simple and standardized measurement techniques.

Crucially, the clinical relevance of these targets is confirmed by our use of a scientifically justified depth boundary. The 2.5 cm cortical mask, applied in our analysis, is derived from the MNI2CPC model [13]. This model, which offers a probabilistic cortex-to-scalp mapping, provides an evidence-based method for determining the spatial relationship between cortical regions and external scalp landmarks. We use this depth as a conservative threshold to ensure all identified targets fall within the estimated effective stimulation reach of non-invasive modalities, giving a strong physical justification to our neuroimaging findings.

Furthermore, the protocol enhances clinical precision by adopting a volumetric targeting approach. Instead of relying on a single pinpoint MNI coordinate, our method uses the cluster’s three-dimensional spatial structure to determine the optimal needle direction and trajectory. This ensures that stimulation or current density is maximized along the entire volume of the pathologically implicated cluster, effectively engaging the full dysfunctional neural assembly identified by the meta-analysis and maximizing therapeutic effect. This pragmatic approach is essential for bridging the gap between large-scale neuroimaging evidence and daily clinical protocols.

### 4.8 Limitations

Although our study provides valuable insights, several limitations warrant consideration. First, the scalp targets we identified are based on published neuroimaging studies; thus, clinical trials are necessary to validate their efficacy and refine stimulation parameters (e.g., intensity, frequency, and duration), which extend beyond the scope of this manuscript. Second, for simplicity, we report only three to nine cortical surface clusters for clinical application. It remains possible that additional and smaller clusters may be integral to the pathophysiology and recovery processes of these disorders. To address this and encourage further research, we have included whole-brain targets in the **Supplementary Tables 1–12**. Third, our primary data acquisition relied on the NeuroStore ecosystem. While this platform aggregates data from extensive sources, relying on a single repository may introduce selection bias, as it may not capture studies indexed solely in other databases. Future studies are needed to replicate and extend these findings by triangulating our data with manual searches of additional databases to further strengthen the validity of the identified targets. Fourth, as the body of literature on this platform continues to expand, the identified targets may evolve over time. We will remain attentive to these developments and update our study as necessary. Fifth, this study lacks formal quality grading for each of the thousands of included publications. While our inclusion criteria ensured basic methodological standards, we did not assess individual study parameters (e.g., sample size, imaging parameters, participant heterogeneity). We relied on the statistical nature of coordinate-based meta-analysis provided by Neurosynth Compose, which identifies areas of high convergence across heterogeneous studies, to mitigate the impact of potential noise from individual lower-quality datasets. Sixth, this meta-analysis method collapses data across all demographics. Mental disorders are highly heterogeneous and may present with different neural correlates across age, sex, and ancestry. Our targets represent a central tendency and may require adjustment for specific populations. Future large-scale meta-analyses, perhaps using more detailed databases, are needed to explore these potential demographic-specific effects. Another significant limitation is the pooling of data from multiple imaging modalities and contrasts. While this approach maximizes statistical power to identify regions of convergence, it may blur specific biological interpretations, such as distinguishing hypo- from hyper-activation. Future fine-grained analyses are needed to provide specific guidance on the direction of modulation.

## 5. Conclusions

We conducted large-scale neuroimaging meta-analyses using Neurosynth Compose to identify cortical targets and propose candidate scalp acupuncture and brain stimulation protocols across nine mental disorders. Our findings highlight both disorder-specific targets and regions frequently involved across conditions, providing a data-driven foundation for future clinical trials. Although this framework is promising, it remains preliminary, and the proposed targets should be prioritized in comparative efficacy studies against traditional protocols. Further clinical validation is needed to translate these neuroimaging-based targets into routine practice.

## Data Availability

All data reported in this paper are provided as part of the submitted article and **Supplementary Material**.

## Abbreviations

WHO, World Health Organization; MRI, magnetic resonance imaging; EEG, electroencephalography; MEG, magnetoencephalography; TMS, transcranial magnetic stimulation; tES, transcranial electrical stimulation; ROIs, regions of interest; SZ, schizophrenia; BD, bipolar disorder; MDD, major depressive disorder; AD, anxiety disorder; OCD, obsessive-compulsive disorder; PTSD, post-traumatic stress disorder; ASD, autism spectrum disorder; ADHD, attention deficit hyperactivity disorder; MNI, Montreal Neurological Institute; L, left; R, right; Bil, bilateral; LPFC, lateral prefrontal cortex; DLPFC, dorsolateral prefrontal cortex; VLPFC, ventrolateral prefrontal cortex; VMPFC, ventromedial prefrontal cortex; DBS, deep brain stimulation.
