# Supplementary material for: Potential Targets for Scalp Acupuncture and Brain Stimulation in Mental Disorders: Evidence From Large-Scale Meta-Analyses
Source: Alpha Psychiatry. 2026 Jun 28;27(3):45940. doi: 10.31083/AP45940 (PMC13339880; doi:10.31083/AP45940)
Supplement: Supplementary file 1 [file 2757-8038-27-3-45940-s1.zip › Supplementary Material.docx]

**Potential scalp acupuncture and brain stimulation targets for common psychiatric disorders: Evidence from large-scale meta-analyses**

**Supplementary Materials**

**Study Protocol for Meta-Analysis**

**1 Data Source**

This study will utilize the Neurosynth Compose platform (https://compose.neurosynth.org), a large-scale and automated meta-analysis tool.

**2 Automated and manual meta-analysis steps**

**2.1 Creating a new project**

To initiate a new meta-analysis, we will first select the New Project option and assign a project name that clearly identifies the target psychiatric disorder.

**2.2 Search & Curate: Import, exclude, and include studies of interest**

The initial phase of a meta-analysis involves searching for relevant studies and compiling them into a final study set suitable for inclusion. We selected the "Simple" curation workflow to initiate an automated literature search and imported studies via Neurostore. This approach enables systematic querying of a comprehensive database containing over 30,000 neuroimaging publications with automatically extracted coordinate data. To maintain temporal consistency across searches, all literature searches for each disorder were conducted within a single day, without restricting the publication date range.

The following search strings were used to identify relevant publications for each disorder. (1) Schizophrenia: “schizophrenia” OR “split personality disorder” OR “paranoid schizophrenia” OR “schizoaffective disorder”; (2) Bipolar Disorder: “bipolar disorder” OR “manic-depressive illness” OR “manic depression” OR “bipolar affective disorder”; (3) Major Depressive Disorder: “major depression” OR “clinical depression” OR “major depressive disorder” OR “unipolar depression” OR “severe depression”; (4) Anxiety Disorder: “anxiety disorder”; (5) Obsessive-Compulsive Disorder: “obsessive-compulsive disorder” OR “OCD” OR “compulsive behavior disorder” OR “obsession disorder”; (6) Post-Traumatic Stress Disorder: “post-traumatic stress disorder” OR “PTSD” OR “combat stress” OR “post-trauma syndrome”; (7) Insomnia: “Insomnia” OR “sleeplessness” OR “sleep deprivation” OR “sleep disorder” OR “difficulty sleeping”; (8) Autism Spectrum Disorder: “autism spectrum disorder” OR “autism” OR “Asperger syndrome” OR “pervasive developmental disorder” OR “childhood disintegrative disorder” OR “high-functioning autism” OR “low-functioning autism”; (9) Attention Deficit Hyperactivity Disorder: “ADHD” OR “attention deficit hyperactivity disorder” OR “attention deficit disorder with hyperactivity” OR “hyperkinesis” OR “attention deficit and disruptive behavior disorders”.

The search results from Neurostore are displayed in the left column of the Curation Board interface. We will perform a systematic manual screening of these results, selecting studies for inclusion according to the predefined criteria outlined below.

A study will be included if it met all of the following criteria: (1) Participants: Involved human participants diagnosed with one of the nine targeted mental disorders; (2) Comparison: Reported a between-group comparison involving the patient group (e.g., patients vs. healthy controls, or pre- vs. post-treatment); (3) Modality: Utilized a neuroimaging modality capable of providing spatial localization, such as functional Magnetic Resonance Imaging (fMRI), Positron Emission Tomography (PET), Single-Photon Emission Computed Tomography (SPECT), Arterial Spin Labeling (ASL), Electroencephalography (EEG), or Magnetoencephalography (MEG); (4) Data: Reported 3D peak coordinates for the between-group comparisons in a standard stereotactic space (Talairach or Montreal Neurological Institute - MNI).

A study will be excluded if it met one or more of the following criteria: (1) Population: Included only healthy participants (e.g., experimental symptom models) or were non-human (animal) studies; (2) Focus: Research unrelated to the targeted mental disorders, or studies where the disorder was not the primary focus (e.g., secondary symptoms or comorbid conditions overshadowed the primary disorder); (3) Data: Does not provide standard-space coordinates for analysis (e.g., region of interest (ROI)-only studies without coordinates); (4) Type: Are neuroimaging meta-analyses, narrative reviews, systematic reviews, or single case reports (to avoid duplication of data); (5) Aim: Are machine learning studies aimed only at predicting treatment response or diagnosis, rather than exploring or reporting neuroimaging findings from group comparisons.

**2.3 Extract & Annotate: Add relevant study data**

At this point, we will create a StudySet containing all of our studies. The goal of this phase is to extract data from the text of studies (coordinates) that will be used in the meta-analysis. For each study, we will designate which analyses (contrasts) to include in our meta-analysis.

**2.4 Specify Meta-Analyses: Create a meta-analysis specification**

During this phase, all the relevant information needed for the meta-analysis will be systematically entered. The subsequent step involves selecting an appropriate meta-analysis algorithm. For the current study, the MKDA Chi-Squared (MKDAChi2) method will be employed. Conceptually, MKDAChi2 tests if there’s evidence of a population level association between the task or psychological construct in our meta-analysis and brain activation (for every voxel). It is equivalent to conducting a chi-squared test of independence for a 2-by-2 table of counts for each voxel, where the binary variables are foci occurrence in the meta-analysis of interest and foci occurrence in the reference set of unselected studies. To control for multiple comparisons, cluster correction was implemented using the False Discovery Rate (FDR) method with an alpha threshold of 0.05.

The meta-analyses were subsequently executed via Google Colab, with the MKDA Chi-Squared workflow producing the statistical uniformity test maps utilized for subsequent target localization in scalp acupuncture protocol development.

**3 Results translation and target identification**

**2.1 Cortical target identification from meta-analysis results**

The FDR-corrected uniformity test map will be first constrained using a standard cortical brain template (within 2.5 cm of the scalp) to identify cortically accessible targets. We will then employ a standardized thresholding procedure in DPABI version 8.1, iteratively increasing the t-value threshold in 0.5 increments until 3-9 clusters meeting voxel count criteria (30-800 voxels per cluster) is identified. This iterative process, consistent with our prior clinical-translational work, serves the dual purpose of focusing on statistically robust clusters and statistically segmenting large, confluent volumes into distinct local maxima. The peak MNI coordinates of these final clusters will be reported using the AAL3 template via xjView, with results visualized on standard brain and head models using Surf Ice and MRIcroGL.

2.2 Scalp mapping and needle application strategies

The identified cortical targets will be translated to the scalp surface using the dual reference frameworks of the International 10-20 EEG system and the WHO Proposed Standard International Acupuncture Nomenclature. Beyond simple surface mapping, the three-dimensional volumetric geometry of each target brain cluster will be analyzed to engineer specific needle insertion vectors and manipulation strategies. This geometry-based approach is designed to maximize the engagement of the underlying neural tissue, ensuring the physical application of scalp acupuncture is spatially optimized for each target’s unique neuroanatomical context.


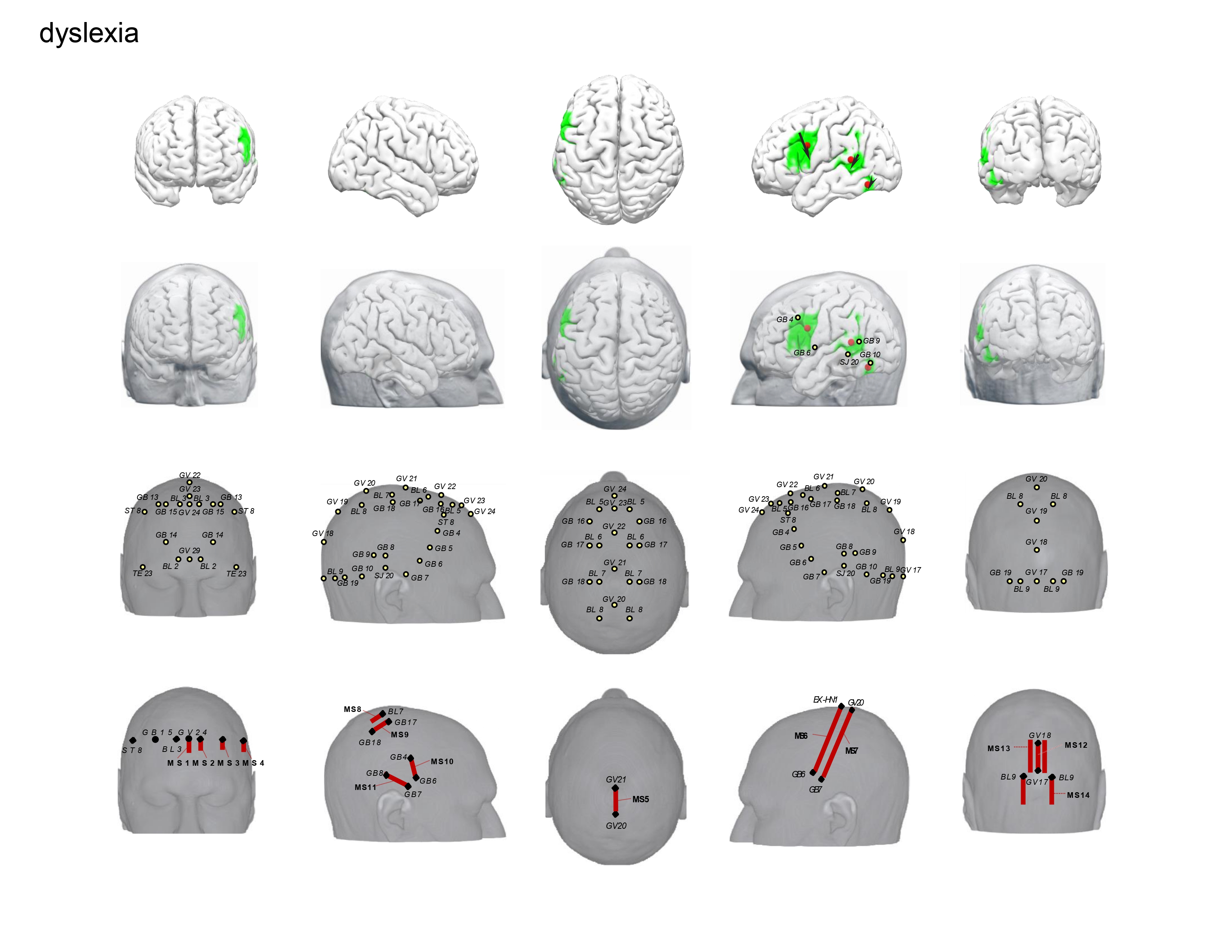


**Supplementary Fig.1. Standard scalp acupoints and lines**.

**
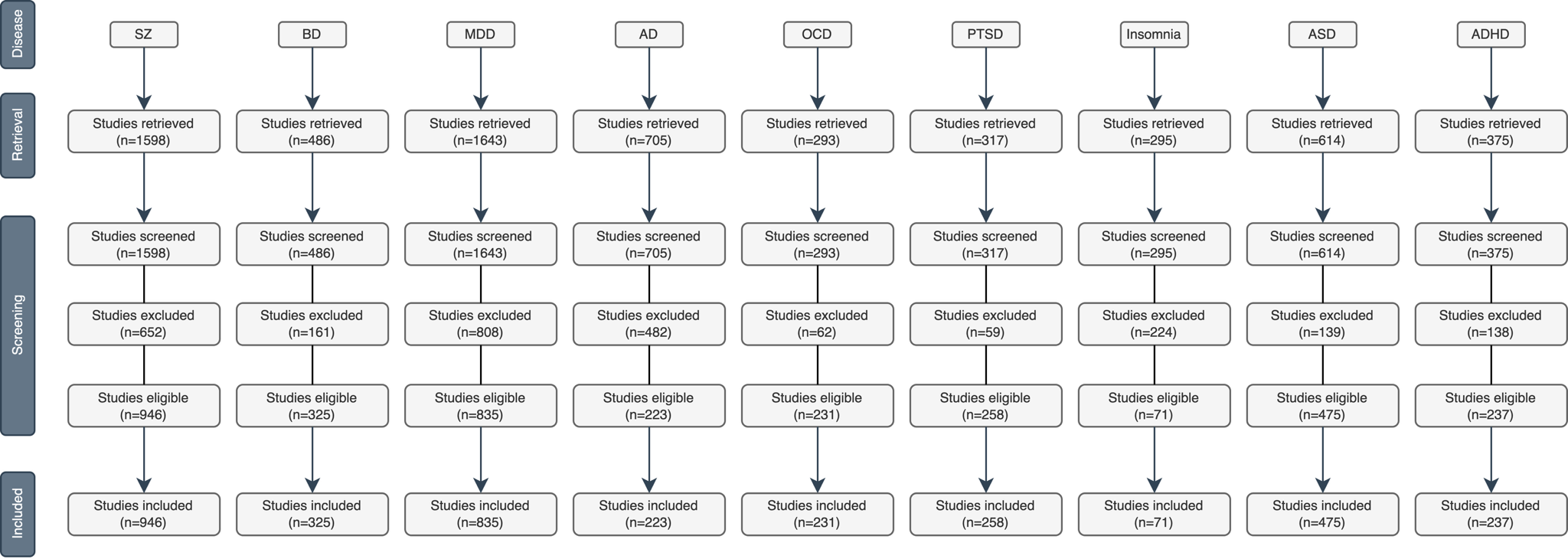
**

**Supplementary Fig.2. Details of the inclusion process. SZ, schizophrenia; BD, bipolar disorder; MDD, major depressive disorder; AD, anxiety disorder; OCD, obsessive-compulsive disorder; PTSD, post-traumatic stress disorder; ASD, autism spectrum disorder; ADHD, attention deficit hyperactivity disorder.**

**Supplementary Table 1 Potential Whole-Brain Targets for Schizophrenia (SZ) Identified Through Meta-Analysis**

| **Targets** | **Number of Voxels** | **T value** | **Peak MNI** | | | **Brain areas of the cluster** |
| --- | --- | --- | --- | --- | --- | --- |
|  |  |  | **X** | **Y** | **Z** |  |
| SZ(2)-1 | 9952 | 18.40 | -42 | 16 | -4 | Frontal_Inf_Tri_Bil/Insula_Bil/Frontal_Inf_Oper_Bil/Precentral_Bil/Temporal_Sup_L/Frontal_Mid_R/Frontal_Inf_Orb_Bil/Putamen_Bil/Caudate_R/Pallidum_R/Thal_VL_L/Temporal_Mid_L/Pallidum_L |
| SZ(2)-2 | 3132 | 16.25 | -4 | 16 | 44 | Supp_Motor_Area_Bil/Frontal_Sup_Medial_Bil/ACC_pre_Bil/Cingulate_Mid_Bil/ACC_sup_Bil/Frontal_Med_Orb_Bil |
| SZ(2)-3 | 181 | 7.44 | 54 | -30 | 2 | Temporal_Sup_R/Temporal_Mid_R |
| SZ(2)-4 | 89 | 7.77 | 48 | -66 | 2 | Temporal_Mid_R |
| SZ(2)-5 | 971 | 12.80 | 0 | -52 | 28 | Precuneus_Bil/Cingulate_Post_Bil |
| SZ(2)-6 | 716 | 9.12 | -34 | -58 | 42 | Parietal_Inf_L/Angular_L/Parietal_Sup_L |
| SZ(2)-7 | 253 | 7.44 | 36 | -50 | 44 | Parietal_Inf_R/Angular_R |

Note: Please refer to the publication for detailed names of brain regions: Rolls E T, Huang C C, Lin C P, et al. Automated anatomical labelling atlas 3[J]. Neuroimage, 2020, 206: 116189. MNI, Montreal Neurological Institute; R, right; L, left; Bil, bilateral.

**Supplementary Table 2 Potential Whole-Brain Targets for bipolar disorder (BD) Identified Through Meta-Analysis**

| **Targets** | **Number of Voxels** | **T value** | **Peak MNI** | | | **Brain areas of the cluster** |
| --- | --- | --- | --- | --- | --- | --- |
|  |  |  | **X** | **Y** | **Z** |  |
| BD(2)-1 | 5830 | 12.15 | 38 | 22 | -6 | Insula_R/Frontal_Inf_Orb_R/Putamen_Bil/Caudate_Bil/Cingulate_Mid_Bil/Hippocampus_Bil/Pallidum_Bil/Amygdala_Bil/Thal_VL_Bil/OFCpost_R/Frontal_Sup_Medial_Bil/ParaHippocampal_R/Thal_MDm_Bil/Supp_Motor_Area_Bil/N_Acc_Bil/Frontal_Inf_Tri_R |
| BD (2)-2 | 1630 | 9.35 | -40 | 20 | 2 | Frontal_Inf_Tri_L/Insula_L/Frontal_Inf_Orb_L/OFCpost_L/Frontal_Inf_Oper_L |
| BD (2)-3 | 1730 | 8.05 | -2 | 40 | 6 | ACC_pre_Bil/Frontal_Med_Orb_Bil/Frontal_Sup_Medial_Bil/ACC_sup_Bil |
| BD (2)-4 | 54 | 4.19 | -28 | 52 | 10 | Frontal_Mid_L/Frontal_Sup_L |
| BD (2)-5 | 145 | 5.50 | 58 | -50 | 14 | Temporal_Mid_R/Temporal_Sup_R |
| BD (2)-6 | 394 | 5.50 | 46 | 40 | 18 | Frontal_Mid_R/Frontal_Inf_Tri_R |
| BD (2)-7 | 144 | 5.17 | 2 | -50 | 26 | Cingulate_Post_Bil |
| BD (2)-8 | 65 | 4.19 | -38 | -56 | 40 | Parietal_Inf_L |
| BD (2)-9 | 224 | 5.17 | 42 | -48 | 44 | Parietal_Inf_R |

Note: Please refer to the publication for detailed names of brain regions: Rolls E T, Huang C C, Lin C P, et al. Automated anatomical labelling atlas 3[J]. Neuroimage, 2020, 206: 116189. MNI, Montreal Neurological Institute; R, right; L, left; Bil, bilateral.

**Supplementary Table 3 Potential Whole-Brain Targets for major depressive disorder (MDD)Identified Through Meta-Analysis**

| **Targets** | **Number of Voxels** | **T value** | **Peak MNI** | | | **Brain areas of the cluster** |
| --- | --- | --- | --- | --- | --- | --- |
|  |  |  | **X** | **Y** | **Z** |  |
| MDD (2)-1 | 17689 | 13.26 | -22 | -8 | -16 | Insula_R/Frontal_Sup_Medial_Bil/Frontal_Mid_L/Frontal_Inf_Tri_Bil/ACC_pre_Bil/Frontal_Mid_R/Frontal_Med_Orb_Bil/Putamen_Bil/Hippocampus_Bil/ACC_sup_Bil/Frontal_Inf_Oper_Bil/Frontal_Inf_Orb_L/Precentral_L/Cingulate_Mid_Bil/Supp_Motor_Area_L/Frontal_Inf_Oper_L/ParaHippocampal_R/Amygdala_Bil |
| MDD (2)-2 | 138 | 5.24 | 26 | -36 | -10 | ParaHippocampal_R/Fusiform_R |
| MDD (2)-3 | 1327 | 8.45 | 4 | -52 | 28 | Precuneus_Bil/Cingulate_Post_Bil |
| MDD (2)-4 | 408 | 5.87 | -50 | -62 | 28 | Angular_L/Occipital_Mid_L |
| MDD (2)-5 | 566 | 6.73 | 50 | -60 | 28 | Angular_R/Parietal_Inf_R |
| MDD (2)-6 | 41 | 4.60 | -42 | -42 | 42 | Parietal_Inf_R |

Note: Please refer to the publication for detailed names of brain regions: Rolls E T, Huang C C, Lin C P, et al. Automated anatomical labelling atlas 3[J]. Neuroimage, 2020, 206: 116189. MNI, Montreal Neurological Institute; R, right; L, left; Bil, bilateral.

**Supplementary Table 4 Potential Whole-Brain Targets for anxiety disorders (AD) Identified Through Meta-Analysis**

| **Targets** | **Number of Voxels** | **T value** | **Peak MNI** | | | **Brain areas of the cluster** |
| --- | --- | --- | --- | --- | --- | --- |
|  |  |  | **X** | **Y** | **Z** |  |
| AD (2)-1 | 976 | 15.33 | -24 | -6 | -16 | Amygdala_L/Hippocampus_L/ParaHippocampal_L/Putamen_L/Pallidum_L |
| AD (2)-2 | 1000 | 12.48 | 24 | -6 | -20 | Hippocampus_R/Amygdala_R/Insula_R/ParaHippocampal_R/Pallidum_R |
| AD (2)-3 | 379 | 7.48 | -4 | 46 | 2 | ACC_pre_L/Frontal_Med_Orb_L/Frontal_Sup_Medial_L |
| AD (2)-4 | 580 | 8.26 | -40 | 14 | 2 | Insula_L/Frontal_Inf_Tri_L/Frontal_Inf_Oper_L |
| AD (2)-5 | 290 | 7.09 | 0 | 20 | 38 | Cingulate_Mid_Bil |

Note: Please refer to the publication for detailed names of brain regions: Rolls E T, Huang C C, Lin C P, et al. Automated anatomical labelling atlas 3[J]. Neuroimage, 2020, 206: 116189. MNI, Montreal Neurological Institute; R, right; L, left; Bil, bilateral.

**Supplementary Table 5 Potential Whole-Brain Targets for obsessive-compulsive disorder (OCD) Identified Through Meta-Analysis**

| **Targets** | **Number of Voxels** | **T value** | **Peak MNI** | | | **Brain areas of the cluster** |
| --- | --- | --- | --- | --- | --- | --- |
|  |  |  | **X** | **Y** | **Z** |  |
| OCD (2)-1 | 194 | 5.06 | 4 | 48 | -12 | Rectus_R/Frontal_Med_Orb_Bil |
| OCD (2)-2 | 6658 | 10.08 | -4 | 12 | 46 | Frontal_Inf_Tri_L/Insula_Bil/Cingulate_Mid_Bil/Supp_Motor_Area_Bil/Caudate_Bil/Frontal_Inf_Orb_Bil/Frontal_Sup_Medial_Bil/Putamen_Bil/ACC_sup_Bil/Pallidum_Bil/Frontal_Inf_Oper_L/Thal_VL_Bil |
| OCD (2)-3 | 143 | 4.68 | 40 | 34 | 28 | Frontal_Mid_R |
| OCD (2)-4 | 76 | 4.68 | 8 | -56 | 32 | Precuneus_R |
| OCD (2)-5 | 40 | 4.30 | -52 | -38 | 38 | Parietal_Inf_L |
| OCD (2)-6 | 197 | 5.06 | 42 | -40 | 42 | SupraMarginal_R/Parietal_Inf_R |
| OCD (2)-7 | 221 | 5.82 | -40 | -54 | 40 | Parietal_Inf_L/Angular_L |

Note: Please refer to the publication for detailed names of brain regions: Rolls E T, Huang C C, Lin C P, et al. Automated anatomical labelling atlas 3[J]. Neuroimage, 2020, 206: 116189. MNI, Montreal Neurological Institute; R, right; L, left; Bil, bilateral.

**Supplementary Table 6 Potential Whole-Brain Targets for post-traumatic stress disorder (PTSD) Identified Through Meta-Analysis**

| **Targets** | **Number of Voxels** | **T value** | **Peak MNI** | | | **Brain areas of the cluster** |
| --- | --- | --- | --- | --- | --- | --- |
|  |  |  | **X** | **Y** | **Z** |  |
| PTSD (2)-1 | 2567 | 12.40 | -20 | -6 | -16 | Amygdala_L/Hippocampus_L/Putamen_L/ParaHippocampal_L/Temporal_Pole_Sup_L/Frontal_Inf_Orb_L/Frontal_Inf_Tri_L/Pallidum_L |
| PTSD (2)-2 | 2632 | 10.66 | 24 | 0 | -16 | Amygdala_R/Insula_R/Hippocampus_R/Frontal_Inf_Tri_R/Frontal_Inf_Oper_R/ParaHippocampal_R/Putamen_R/Frontal_Inf_Orb_R/Pallidum_R |
| PTSD (2)-3 | 3549 | 10.00 | -4 | 48 | -4 | Frontal_Sup_Medial_Bil/ACC_pre_Bil/ACC_sup_Bil/Frontal_Med_Orb_L/Cingulate_Mid_Bil/Supp_Motor_Area_Bil |
| PTSD (2)-4 | 305 | 5.69 | 0 | -48 | 28 | Cingulate_Post_Bil/Precuneus_Bil |
| PTSD (2)-5 | 37 | 3.99 | -48 | 12 | 28 | Frontal_Inf_Oper_L/Precentral_L |

Note: Please refer to the publication for detailed names of brain regions: Rolls E T, Huang C C, Lin C P, et al. Automated anatomical labelling atlas 3[J]. Neuroimage, 2020, 206: 116189. MNI, Montreal Neurological Institute; R, right; L, left; Bil, bilateral.

**Supplementary Table 7 Potential Whole-Brain Targets for insomnia Identified Through Meta-Analysis**

| **Targets** | **Number of Voxels** | **T value** | **Peak MNI** | | | **Brain areas of the cluster** |
| --- | --- | --- | --- | --- | --- | --- |
|  |  |  | **X** | **Y** | **Z** |  |
| insomnia-1 | 57 | 3.73 | -38 | 10 | -20 | Temporal_Pole_Sup_L/Insula_L |
| insomnia-2 | 75 | 3.74 | 26 | 8 | 0 | Putamen_R/Pallidum_R |
| insomnia-3 | 44 | 2.83 | -52 | 6 | 22 | Precentral_L/Frontal_Inf_Oper_L |
| insomnia-4 | 52 | 3.74 | -62 | -50 | 32 | SupraMarginal_L |
| insomnia-5 | 527 | 6.51 | 4 | 18 | 54 | Supp_Motor_Area_Bil/Frontal_Sup_Medial_Bil |
| insomnia-6 | 30 | 3.74 | -54 | -30 | 40 | Parietal_Inf_L |
| insomnia-7 | 43 | 3.74 | 54 | -50 | 46 | Parietal_Inf_R |

Note: Please refer to the publication for detailed names of brain regions: Rolls E T, Huang C C, Lin C P, et al. Automated anatomical labelling atlas 3[J]. Neuroimage, 2020, 206: 116189. MNI, Montreal Neurological Institute; R, right; L, left; Bil, bilateral.

**Supplementary Table 8 Potential Whole-Brain Targets for autism spectrum disorder (ASD) Identified Through Meta-Analysis**

| **Targets** | **Number of Voxels** | **T value** | **Peak MNI** | | | **Brain areas of the cluster** |
| --- | --- | --- | --- | --- | --- | --- |
|  |  |  | **X** | **Y** | **Z** |  |
| ASD (2)-1 | 3807 | 8.49 | 54 | -60 | 4 | Temporal_Mid_R/Temporal_Sup_R/Fusiform_R/Angular_R/Temporal_Inf_R/SupraMarginal_R/Occipital_Inf_R |
| ASD (2)-2 | 543 | 8.05 | 26 | -4 | -16 | Amygdala_R/Hippocampus_R/ParaHippocampal_R |
| ASD (2)-3 | 6556 | 8.72 | -36 | 18 | 0 | Insula_L/Temporal_Mid_L/Frontal_Inf_Tri_L/Precentral_L/Parietal_Inf_L/Temporal_Sup_L/Fusiform_L/Frontal_Inf_Oper_L/Occipital_Mid_L/Frontal_Inf_Orb_L/Postcentral_L/Occipital_Inf_L |
| ASD (2)-4 | 346 | 6.47 | -24 | -2 | -18 | Amygdala_L/Hippocampus_L |
| ASD (2)-5 | 2536 | 7.15 | -4 | 12 | 50 | Frontal_Sup_Medial_Bil/Supp_Motor_Area_Bil/ACC_pre_Bil/Frontal_Sup_Medial_R/Frontal_Med_Orb_Bil/ACC_sup_Bil/Cingulate_Mid_Bil/ |
| ASD (2)-6 | 39 | 3.60 | 22 | -26 | -14 | ParaHippocampal_R |
| ASD (2)-7 | 2829 | 10.08 | 38 | 22 | 2 | Insula_R/Frontal_Inf_Oper_R/Frontal_Inf_Tri_R/Precentral_R/Frontal_Inf_Orb_R/Frontal_Mid_R/Frontal_Sup_R/Putamen_R/ |
| ASD (2)-8 | 157 | 4.90 | -22 | -92 | 6 | Occipital_Mid_L |
| ASD (2)-9 | 115 | 4.01 | 10 | -20 | 10 | Thal_PuA_R/Thal_VL_R |
| ASD (2)-10 | 1121 | 7.37 | -4 | -64 | 32 | Precuneus_L/Precuneus_/Cingulate_Post_ |
| ASD (2)-11 | 37 | 4.01 | 24 | -64 | 54 | Parietal_Sup_R |

Note: Please refer to the publication for detailed names of brain regions: Rolls E T, Huang C C, Lin C P, et al. Automated anatomical labelling atlas 3[J]. Neuroimage, 2020, 206: 116189. MNI, Montreal Neurological Institute; R, right; L, left; Bil, bilateral.

**Supplementary Table 9 Potential Whole-Brain Targets for attention deficit hyperactivity disorder (ADHD) Identified Through Meta-Analysis**

| **Targets** | **Number of Voxels** | **T value** | **Peak MNI** | | | **Brain areas of the cluster** |
| --- | --- | --- | --- | --- | --- | --- |
|  |  |  | **X** | **Y** | **Z** |  |
| ADHD (2)-1 | 12056 | 9.89 | -34 | 18 | -4 | Insula_Bil/Frontal_Mid_R/Putamen_Bil/Cingulate_Mid_Bil/Frontal_Inf_Oper_R/Supp_Motor_Area_Bil/Frontal_Inf_Tri_RCaudate_Bil/Pallidum_Bil/Frontal_Inf_Tri_L/Frontal_Inf_Orb_Bil/Precentral_Bil/Frontal_Sup_Medial_L/N_Acc_Bil/Frontal_Sup_Bil/Thal_VL_L |
| ADHD (2)-2 | 1708 | 6.32 | -2 | 48 | 2 | ACC_pre_Bil/Frontal_Sup_Medial_Bil/Frontal_Med_Orb_Bil ACC_sup_Bil/ACC_sub_Bil |
| ADHD (2)-3 | 54 | 3.52 | -46 | -64 | -6 | Temporal_Inf_L/Occipital_Inf_L |
| ADHD (2)-4 | 1436 | 4.93 | 44 | -48 | 42 | Parietal_Inf_R/SupraMarginal_R/Precuneus_R/Angular_R |
| ADHD (2)-5 | 467 | 4.58 | -8 | -64 | 18 | Calcarine_L/Precuneus_L/Cingulate_Post_L |
| ADHD (2)-6 | 517 | 4.23 | -44 | 30 | 20 | Frontal_Inf_Tri_L/Precentral_L/Frontal_Mid_L |
| ADHD (2)-7 | 1298 | 5.97 | -38 | -44 | 44 | Parietal_Inf_L/Parietal_Sup_L/SupraMarginal_L/Angular_L |

Note: Please refer to the publication for detailed names of brain regions: Rolls E T, Huang C C, Lin C P, et al. Automated anatomical labelling atlas 3[J]. Neuroimage, 2020, 206: 116189. MNI, Montreal Neurological Institute; R, right; L, left; Bil, bilateral.

**Supplementary Table 10 Different naming of the acupoints involved in these protocols**

|  | **WHO Standard Code** | **Chinese Name (Pinyin)** | **Japanese Name (Kanji, Reading)** | **Korean Name (Hangul, Reading)** |
| --- | --- | --- | --- | --- |
| 1 | BL8 | 络却 (Luoque) | 絡却 (らっきゃく, Rakkaku) | 락각 (락각, Ragag) |
| 2 | GB10 | 浮白 (Fubai) | 浮白 (ふはく, Fuhaku) | 부백 (부백, Bubek) |
| 3 | GB14 | 阳白 (Yangbai) | 陽白 (ようはく, Yōhaku) | 양백 (양백, Yangbaek) |
| 4 | GB15 | 头临泣 (Toulinqi) | 頭臨泣 (とうりんきゅう, Tōrinkiyū) | 두임읍 (두임읍, Duimeup) |
| 5 | GB17 | 正营 (Zhengying) | 正営 (せいえい, Sei'ei) | 정영 (정영, Jeongyeong) |
| 6 | GB18 | 承灵 (Chengling) | 承霊 (じょうれい, Jōrei) | 승령 (승령, Seungnyeong) |
| 7 | GB19 | 脑空 (Naokong) | 腦空 (のうくう, Nōkū) | 뇌공 (뇌공, Noegong) |
| 8 | GB4 | 颔厌 (Hanyan) | 頷厭 (がんえん, Gan'en) | 함염 (함염, Hamyeom) |
| 9 | GB5 | 悬颅 (Xuanlu) | 懸顱 (けんろ, Kenro) | 현로 (현로, Hyeonno) |
| 10 | GB6 | 悬厘 (Xuanli) | 懸厘 (けんり, Kenri) | 현리 (현리, Hyeonri) |
| 11 | GB7 | 曲鬓 (Qubin) | 曲鬢 (きょくびん, Kyokubin) | 곡빈 (곡빈, Gokbin) |
| 12 | GB8 | 率谷 (Shuaigu) | 率谷 (そっこく, Sokkoku) | 솔곡 (솔곡, Solgok) |
| 13 | GB9 | 天冲 (Tianchong) | 天衝 (てんしょう, Tenshō) | 천충 (천충, Cheonchung) |
| 14 | GV20 | 百会 (Baihui) | 百会 (ひゃくえ, Hyakue) | 백회 (백회, Baekhoe) |
| 15 | GV22 | 囟会 (Xinhui) | 囟会 (しんえ, Shinhui) | 신회 (신회, Sinhui) |
| 16 | GV24 | 神庭 (Shenting) | 神庭 (しんてい, Shintei) | 신문 (신문, Sinmun) |
| 17 | GV29 | 印堂 (Yintang) | 印堂（いんどう, Indō） | 印당 (인당, Indang) |
| 18 | SJ20 | 角孙 (Jiaosun) | 角孫 (かくそん, Kakuson) | 각손 (각손, Gakson) |
| 19 | ST8 | 头维 (Touwei) | 頭維 (とうい, Tōi) | 두유 (두유, Duyu) |
| 20 | TE23 | 丝竹空 (Sizhukong) | 絲竹空 (しちくくう, Shichikukū) | 사죽공 (사죽공, Sajukgong) |

**Supplementary Table 11 Comparison of lesion- and stimulation-derived symptom circuits with meta-analytic findings across psychiatric disorders: cortical brain regions**

|  | **Causal Circuit Regions (Lesion-/Stimulation-derived)** | **Meta-analysis Regions** |
| --- | --- | --- |
| **Schizophrenia** | *Retrosplenial Cortex*  *Superior Temporal Gyrus* | *Temporal_Sup_R*  *Precuneus_Bil*  *Cingulate_Post_Bil*  Frontal_Inf_Tri_Bil  Insula_Bil  Frontal_Inf_Oper_Bil  Supp_Motor_Area_Bil  Frontal_Sup_Medial_Bil  ACC_pre_Bil  Temporal_Mid_R  Parietal_Inf_ Bil  Angular_ Bil  Parietal_Sup_L |
| **Major depressive disorder** | *Dorsolateral Prefrontal Cortex_L (-53, 41, 15)*  *Dorsolateral Prefrontal Cortex_R (48, 38, 23)*  *Inferior Frontal Gyrus_L (-46, 9, 31)*  *Inferior Frontal Gyrus_R (46, 4, 35)*  *Ventromedial Prefrontal Cortex (-6, 58, 10)*  Intraparietal Sulcus_L (-33, -53, 46)  Intraparietal Sulcus_R (34, -51, 46)  Extrastriate Visual Cortex_L (-57, -50, -8)  Subgenual Cingulate Cortex (8, 24, -4) | *Middle frontal gyrus_L (-32, 52, 18)*  *Middle frontal gyrus_R (38, 42, 24)*  *Inferior frontal gyrus _L (-42, 38, 6)*  *Inferior frontal gyrus _R (56, 16, 10) (48, 36, 0)*  *Medial prefrontal gyrus_Bil (-4, 58, 0)*  Angular gyrus_R (50, -60, 28)  Angular gyrus_L (-50, -62, 28)  Precentral gyrus_L (-48, 6, 28) |
| **Post-traumatic stress disorder** | *medial Prefrontal Cortex*  Anterolateral Temporal Lobe  Dorsolateral Prefrontal Cortex | *Frontal_Sup_Medial_Bil*  Insula_R  Cingulate_Post_Bil  Frontal_Inf_Oper_L |
| **Obsessive-compulsive disorder** | *Right Frontal Pole, Frontal Medial & Orbital Cortices*  *(14, 44, -14)*  Left Frontal Pole, Frontal Medial & Orbital Cortices  (-22, 54, -14) | *Lateral / posterior orbital gyrus_R (52, 20, -8)*  middle frontal gyrus_ R (42, 14, 36) (40, 34, 28)  Inferior frontal gyrus_L (-42, 26, 24)  Precentral gyrus_ L (-50, 18, -4)  Middle / inferior frontal gyrus_L (-38, 2, 46)  Angular gyrus_ L (-42, -56, 42)  Supramarginal gyrus_R (46, -40, 48) |
| **Anxiety disorders** | *Right Lateral Parietal Lobe (47, -59, 32)*  Right Superior Frontal Gyrus (22, 31, 42) | *Inferior parietal gyrus_ R (48, -52, 38)*  Superior frontal gyrus_L (-6, 54, 30)  Middle frontal gyrus_ L (-34, 38, 26)  Precentral gyrus_R (48, 10, 36)  Inferior frontal gyrus_L (-50, 18, 2)  Middle temporal gyrus_L (-62, -40, 1) |

Note: Italicized regions indicate potential overlap between the two methods.

**Supplementary Table 12 Comparison of lesion- and stimulation-derived symptom circuits with meta-analytic findings across psychiatric disorders: subcortical brain regions**

|  | **Causal Circuit Regions (Lesion-/Stimulation-derived)** | **Meta-analysis Regions** |
| --- | --- | --- |
| **Schizophrenia** | Posterior Subiculum  Ventral Tegmental Area  *Mediodorsal Thalamus & Midline Thalamic Nuclei*  Right Mediodorsal Thalamus | *Thal_VL_* *Bil* |
| **Major depression** | - | Amygdala_ L (-22, -8, -16)  ParaHippocampal_R (26, -36, -10) |
| **Post-traumatic stress disorder** | *Amygdala*  *Hippocampus*  Tapetum of the Corpus Callosum | *Amygdala_ Bil*  *Hippocampus_ Bil*  Putamen_Bil |
| **Obsessive-compulsive disorder** | Right Accumbens, Caudate & Putamen (16, 18, -8)  Left Accumbens, Caudate & Putamen (-20, 18, -8) | - |
| **Anxiety disorders** | - | Amygdala_ L (-24, -6, -16)  Amygdala_ R (24, -6, -20) |

Note: Italicized regions indicate potential overlap between the two methods.
